# Exploring Hyperoxia Effects in Cancer—From Perioperative Clinical Data to Potential Molecular Mechanisms

**DOI:** 10.3390/biomedicines9091213

**Published:** 2021-09-13

**Authors:** Anca Irina Ristescu, Crina Elena Tiron, Adrian Tiron, Ioana Grigoras

**Affiliations:** 1 Department of Anaesthesia and Intensive Care, School of Medicine, “Grigore T. Popa” University of Medicine and Pharmacy, 700115 Iasi, Romania; anca.ristescu@umfiasi.ro (A.I.R.); ioana.grigoras@umfiasi.ro (I.G.); 2 Department of Anaesthesia and Intensive Care, Regional Institute of Oncology, 700483 Iasi, Romania; 3 TRANSCEND Research Centre, Regional Institute of Oncology, 700483 Iasi, Romania; transcendctiron@iroiasi.ro

**Keywords:** hyperoxia, reactive oxygen species, cancer progression, epithelial mesenchymal transition, oxidative stress, BDNF

## Abstract

Increased inspiratory oxygen concentration is constantly used during the perioperative period of cancer patients to prevent the potential development of hypoxemia and to provide an adequate oxygen transport to the organs, tissues and cells. Although the primary tumours are surgically removed, the effects of perioperative hyperoxia exposure on distal micro-metastases and on circulating cancer cells can potentially play a role in cancer progression or recurrence. In clinical trials, hyperoxia seems to increase the rate of postoperative complications and, by delaying postoperative recovery, it can alter the return to intended oncological treatment. The effects of supplemental oxygen on the long-term mortality of surgical cancer patients offer, at this point, conflicting results. In experimental studies, hyperoxia effects on cancer biology were explored following multiple pathways. In cancer cell cultures and animal models, hyperoxia increases the production of reactive oxygen species (ROS) and increases the oxidative stress. These can be followed by the induction of the expression of Brain-derived neurotrophic factor (BDNF) and other molecules involved in angiogenesis and by the promotion of various degrees of epithelial mesenchymal transition (EMT).

## 1. Background

The incidence of the main types of solid tumours is continuously rising around the world, despite increasing public awareness on modifiable risk factors and the implementation of preventive strategies, but this is also related to advances in screening programs and diagnostic tools. At the same time, the number of oncologic patients undergoing diagnostic, staging, curative, reconstructive or palliative surgical interventions is following the same demographic. It was estimated that, in 2030, there will be 45 million cancer surgeries and, from 22 million new cancer cases, about 17 million patients will need surgical interventions [1].

Despite the development of a more targeted and personalised multimodal treatment, cancer mortality remains high. In the USA, during 2020, a COVID-19 pandemic year, cancer was responsible for almost 600,000 casualties, representing the second leading cause of death after heart disease [2]. The main cause of death is cancer progression, the occurrence of distal metastasis or cancer recurrence, as the vast majority of patients are now successfully treated for primary tumours, with 64% of them having more than 5 years survival [3].

Most cancer patients will need surgery at some time during the cancer care continuum. The perioperative period is currently considered a highly vulnerable time frame in all oncologic patients. Multiple surgical and anaesthetic factors, acting at the systemic level, on the tumoral microenvironment and/or directly on cancer cells, can increase the risk of both postoperative complications and cancer progression [4,5,6]. Onco-anaesthesia, a recently emerged sub-speciality, aims to improve both short-term surgical outcome and also long-term oncological outcome, through the identification of the anaesthetic related factors that can protect from, or can be related to, cancer recurrence and metastasis [7]. While anaesthetic techniques (general versus loco-regional anaesthesia and inhalation versus total intravenous anaesthesia) and drugs (mainly propofol, sevoflurane, lidocaine, opioids) were broadly studied during the last few years [8,9,10,11,12,13], the role of perioperative high inspiratory oxygen concentration exposure is currently not well defined.

In cancer patients receiving general anaesthesia for surgery, increased inspiratory oxygen concentration is commonly used both intraoperatively and, for a variable time period, postoperatively, in order to prevent the potential development of hypoxemia and to ensure an adequate oxygen transport to the organs, tissues and cells. For a long time considered as a useful and harmless intervention, oxygen therapy has been under scrutiny in recent years due to the accumulation of data regarding its deleterious effects in critically ill patients [14] and many areas of acute medicine (stroke, acute myocardial infarction, cardiac arrest) [15,16,17,18,19]. In critically ill patient care, current data does not support a benefit from supranormal oxygen delivery. A meta-analysis of 19 clinical studies showed that hyperoxaemia was associated with increased in-hospital mortality [20]. A large observational multicentric study, collecting almost 300,000 arterial blood gas analysis from more than 14,000 critically ill patients, demonstrated a linear relationship between hyperoxemia and mortality, with both duration and severity adversely affecting the outcome [21]. A recent systematic review of 16,000 critically ill patients suggested that hyperoxia exposure might be associated with increased hospital mortality [22]. The British clinical practice guideline for oxygen therapy in acutely ill medical patients recommends to stop supplemental oxygen therapy when SpO_2_ reaches 96% and not to start oxygen therapy at or above 93% oxygen saturation in patients with acute stroke or myocardial infarction (strong recommendations) [23].

During the perioperative care of an oncologic patient, the anaesthetist may currently choose between different types of anaesthesia techniques (general anaesthesia versus regional or neuraxial anaesthesia) and can also choose between various anaesthetic and analgesic agents (inhalation versus intravenous anaesthesia). However, oxygen, the most commonly used perioperative drug, cannot be replaced. The optimal dose and duration for oxygen therapy in surgical cancer patients is not clearly established.

The purpose of this narrative review is to summarize and to critically review the currently available clinical and experimental evidence regarding the potential effects of hyperoxia exposure on cancer progression. We further explored the relationship between hyperoxia and reactive oxygen species (ROS) production, the consecutive ROS signalling molecular pathways and the connection with angiogenesis and epithelial mesenchymal transition (EMT) as key biological processes that may foster and sustain cancer growth.

## 2. Hyperoxia Effects on Surgical Cancer Patients—Clinical and Experimental Data

There is an ongoing debate in the literature regarding the potential benefits or harms associated with the use of increased inspiratory oxygen concentration during the perioperative period. The usefulness claimed by hyperoxia supporters consists of the reduction of surgical site infections (SSI) in intubated surgical patients [24], the antiemetic effects and a theoretical margin of safety in case of intraoperative emergencies [25]. On the other hand, harmful effects of hyperoxia can occur at multiple levels.

### 2.1. Short-Term Effects of Hyperoxia in Surgical Patients

Hyperoxia exposure in surgical patients may have potentially deleterious respiratory, cardiovascular and cerebral effects and was correlated in clinical and experimental studies with an increased risk of postoperative pulmonary complications and major adverse cardiac events (MACE).

#### 2.1.1. Respiratory Effects

As the lung is the first and the most exposed organ to high inspiratory oxygen concentration, multiple respiratory effects of hyperoxia have been described—Hyperoxic Acute Lung Injury (HALI), characterized by biochemical damage of the tracheobronchial mucosa and alveoli, decreased surfactant production, capillary endothelium alteration and increased production of inflammatory cytokines [26,27]; absorption atelectasis and the inhibition of hypoxic pulmonary vasoconstriction [28].

Hyperoxia exposure effects postoperative pulmonary complications. Multiple clinical and experimental studies have tried to evaluate the consequences of hyperoxia on the postoperative pulmonary function and/or complications. A post-hoc analysis [29] of nearly 5000 surgical patients enrolled in a cohort study [30] showed that perioperative exposure to 80% oxygen does not worsen pulmonary outcome. Additionally, a recent meta-analysis [31] showed that the administration of 80% vs. 30% oxygen was not associated with increased postoperative pulmonary complications. However, this meta-analysis does not include one important retrospective study analysing data of almost 74,000 surgical patients submitted to non-cardiothoracic interventions [32]. The authors of this study found that high intraoperative oxygen concentration was associated, in a dose-dependent manner, with major respiratory complications and with increased 30-day mortality [32]. 

#### 2.1.2. Cardiovascular Effects

Hyperoxia exposure cardiovascular consequences include systemic vasoconstriction with increased systemic vascular resistance, reduced cerebral and coronary blood flow, reduced cardiac output and pulmonary vasodilation with decrease pulmonary vascular resistance [33,34,35].

The clinical relevance of the cardiovascular effects of perioperative hyperoxia exposure was explored on the postoperative risk of myocardial ischemia. Myocardial injury after noncardiac surgery (MINS) is a frequent postoperative complication, resulting from an imbalance between oxygen myocardial supply and demand. A secondary analysis of data from a large, multicentre, randomized controlled trial, including abdominal surgical patients exposed to either 30% or 80% oxygen intra and postoperatively (PROXI trial), concluded that perioperative hyperoxia might increase the risk of myocardial infarction and cardiovascular diseases [36]. In acute myocardial infarction patients, hyperoxia can increase the production of reactive oxygen species and can aggravate myocardial dysfunction by reducing the myocardial perfusion [37]. Therefore, for the management of acute myocardial infarction patients, current recommendations include tailoring oxygen therapy in order to avoid hyperoxemia [23,38].

#### 2.1.3. Cerebral Effects

The reduction of the cerebral flow and neurotoxicity were described after hyperoxia exposure [39]. The clinical relevance of these effects was not clearly established, as a recent post hoc follow-up study including abdominal surgical patients exposed to 30% vs. 80% oxygen, found no difference in the incidence of stroke or transient cerebral ischemia [40].

Any postoperative complication in patients submitted to major oncologic surgery may have important consequences. On the one hand, adverse postoperative events are associated with decreased overall or decreased recurrence-free survival, as has been demonstrated in recent publications for different types of cancers [41,42]. It has also been shown that the postoperative period is a more important determinant of survival after major surgery than the preoperative risk factors [43]. A possible explanation relating the occurrence of the postoperative complications with cancer progression and outcome could be the amplification of surgical-induced systemic inflammation followed by immune suppression. On the other hand, the development of major complications after cancer surgery can delay or contraindicate subsequent oncological treatments (chemo-, radio-, immune-therapy) [44]. The concept of return to intended oncologic treatment (RIOT) was recently proposed [45], as a novel quality metric for oncological anaesthesia and surgery. Both anaesthetic and surgical strategies, developed in order to reduce potentially avoidable postoperative complications, to enhance recovery and to improve RIOT in oncologic patients with different types of solid tumours, were investigated and proposed [46,47,48].

### 2.2. Long-Term Effects of Hyperoxia on Surgical Cancer Patients

Data regarding the effects of increased inspiratory oxygen concentration exposure and consecutive hyperoxemia on cancer patient outcome come from the secondary analysis of three clinical trials, primarily investigating hyperoxia exposure effects on the development of surgical site infections. 

In the first large, multicentre, randomized controlled trial, Supplemental Oxygen and Complications After Abdominal Surgery, the PROXI trial, including 1400 abdominal surgical patients, the authors found that 80% versus 30% oxygen exposure did not decrease the incidence of surgical site infections (SSI) [49]. In a follow-up analysis of this study [50], the authors reported, in a subgroup of 352 surgical cancer patients, an increased long-term mortality (HR 1.45, 95% CI, 1.10–1.90) with 45% increased hazard ratio over a median of 2.3 years. The authors found that the increased long-term mortality in the 80% oxygen group appeared to be statistically significant in patients undergoing cancer surgery but not in non-cancer patients. Among the possible mechanisms involved, the authors proposed hyperoxia induced angiogenesis that can favour tumoral growth, increased erythropoietin release and DNA damage by reactive oxygen species. The risk of new or recurrent cancer after 80% versus 30% perioperative inspiratory oxygen concentration during abdominal surgery was further assessed in 1377 patients, who completed a median 3.9 year follow-up period [51]. This analysis showed that new cancers occurred at a similar rate, but the cancer-free survival time was significantly shorter in the 80% oxygen group. However, earlier recurrence was insufficient to explain the excess mortality in cancer patients. 

The second published study [52] consisted of a long-term mortality analysis of a subset of patients previously included in two trials investigating supplemental oxygen effects on surgical wound infection [53,54]. In 927 patients scheduled for elective colorectal surgery and randomly assigned to receive 30% or 80% oxygen perioperatively (during general anaesthesia and for up to 2 h postoperative), the mortality was no different. Importantly, for the included 451 colorectal cancer patients, the outcome was similar in both 30% and 80% oxygen groups, with a hazard ratio of 0.85 (95% CI, 0.64–1.1) [52].

The third published study is a large, single-centre, multiple crossover cluster trial including 5000 colorectal surgeries performed on 4088 adults patients. The authors tested the effects of 80% versus 30% oxygen concentration on a composite of 30-day deep and organ-space SSIs, healing-related wound complications and mortality, and found that supplemental oxygen does not prevent major infections and healing-related complications after major intestinal surgery [30]. A recent post-hoc analysis of this study, including 3400 operations performed on 2800 patients, investigated patient long-term mortality [55]. After a median of 3 years, the incidence of death was no different in the 80% versus the 30% oxygen group (13% vs. 14%, 95% CI = 0.78–1.13; *p* = 0.493). The analysis of 995 colorectal cancer patients also showed that 80% inspired oxygen did not influence long-term mortality [55]. 

Thus, as shown in Table 1, clinical studies testing supplemental oxygen effects on the long-term mortality of surgical cancer patients currently offer conflicting results. These can be related to the heterogeneity of the patients’ demographics, of the surgical interventions (emergency versus elective, major, intermediate or minor surgery) and of the hyperoxia exposure period. 

## 3. Potential Molecular Mechanisms Exploring Hyperoxia Effects on Cancer Progression

Hyperoxia effects on cancer biology were explored following multiple pathways, using both in vitro cancer cell cultures and in vivo tumoral animal models. Experimental data support clinical evidences demonstrating that hyperoxia, mainly if prolonged, can induce lung injury and cerebral damage [56,57,58,59,60,61,62], and this can be counteracted by down-modulation of Akt [63] or by low-dose vitamin D [64] or aspirin [65]. Moreover, hyperoxia diminishes protein synthesis [66], and high levels of reactive oxygen species trigger expression of several microRNAs in cardiac and pulmonary diseases, as recently reviewed [67,68,69].

### 3.1. ROS Production and Oxidative Stress

Hyperoxia never occurs during natural circumstances. Thus, while normal tissues and cells have extensive adaptive mechanisms to hypoxemia, they have limited protection against hyperoxia. In order to maintain normal cellular functions, a highly regulated balance between oxidant and antioxidant molecular activity emerged during evolution. High concentration oxygen exposure results in increased ROS formation and oxidative stress, overwhelming the antioxidant mechanisms capacity and producing DNA damage, protein damage and lipid peroxidation [70]. ROS are constantly generated and eliminated in biological systems via several regulatory pathways. ROS production is modulated by endogenous and exogenous factors, including oxygen, environmental stressors or ionizing radiation in a dose dependent manner. While intrinsic levels of ROS are involved in the maintenance of cellular homeostasis, high levels of ROS can be toxic to cells. Thus, oxidative stress may lead to cell damage or cell death by apoptosis or necrosis. The severity of cell disturbances depends on the cumulative oxygen dose (concentration and duration of exposure) and on cell susceptibility. At the same time, when a cell experiences high stress levels, it activates autocrine and paracrine mechanisms to protect itself and other cells. In clinical practice, these subtle effects are almost silent and undetected during the postsurgical recovery but may have long-term outcome consequences.

In surgical patients, a recent study confirmed that intraoperative and postoperative hyperoxia exposure (administration of FiO_2_ = 0.80 in anesthetized patients undergoing abdominal surgery) alters the redox equilibrium at 24 h after surgery, demonstrated by increased lipid peroxidation and decreased antioxidant barrier strength [71].

In many human solid cancers, an important difference in oxygen concentration between the peripheral and the central tumoral cells has been described [72,73]. This variation, mainly explained by the rapid tumoral growth related to the peripheral distribution of blood vessels and abnormal angiogenesis, leads to a hypoxic environment inside the tumoral core. The molecular mechanisms triggered by tumoral hypoxia have been widely investigated. The decreased oxygen availability (intra-tumour hypoxia) will trigger an adaptive response, initially represented by up-regulation and increased levels of the Hypoxia Inducible Factor-1α (HIF-1α) protein. In addition, major genetic and epigenetic alterations already present on the cancer cells can further increase HIF-1α activity [72]. HIF-1α accumulates and induces, at the nuclear level, the transcription of multiple target genes. This process is followed by the synthesis of different proteins involved in angiogenesis, such as Vascular endothelial growth factor A (VEGF-A), in metabolic shift, such as glucose transporters GLUT-1, GLUT-3, in pH adaptation, such as carbonic anhydrase enzymes CA-9, CA-12, and also in migration, invasion, proliferation and metastasis, such as Insulin-like growth factor (IGF-2) and E-Cadherin [73].

Various regimens of normobaric or hyperbaric high oxygen concentrations are used to re-sensitize chemoresistant cancer cells [74,75], as a treatment of various cancer types [76,77,78], as a modulator of immune system anti-cancer responses [79] or in wound healing and neuroprotection [80,81,82].

### 3.2. Hyperoxia and the Immune System

Hypoxia has the ability to regulate immunosuppressive mechanisms that involve myeloid-derived suppressor cells (MDSCs), tumour-associated macrophages (TAMs), regulatory T-cells (Treg cells) and immune checkpoint pathways, such as the programmed cell death-1 (PD-L1). Using a murine model of triple negative breast cancer, Qian et al. demonstrated that long-time respiratory hyperoxia exposure, 60% O_2_ concentration administered continuously for 21 days can reverse immunosuppression by regulating MDSCs and PD-L1 expression [79]. Moreover, Hatfield et al. demonstrated, in a murine model of lung cancer, that increased inspiratory oxygen concentrations decreased immunosuppressive molecules, such as transforming growth factor–β (TGF-β), weakened immunosuppression by regulatory T cells and improved lung tumour regression and long-term survival in mice [83]. In a non-cancer model, Kumar et al. demonstrated that neonatal hyperoxia alters the adaptive immune response in adult mice, a mechanism which increase the risk for susceptibility to infection in premature infants [84].

The interaction of hyperoxia with the immune system will require additional investigations regarding the oxygen concentration, exposure length and patient status as there is another piece of evidence that indicates that hyperoxia does not exert immunologic effects in murine and human experimental setups [85]. The exposure of mice for 2.5 h or of healthy volunteers for 3.5 h to 100% oxygen does not affect the inflammatory response induced by administration of endotoxin. The data suggest that short-term hyperoxia has different effects on cancer patients compared with healthy volunteers.

### 3.3. Angiogenesis and Epithelial Mesenchymal Transition (EMT)

In an in vitro experimental study testing xenon- and sevoflurane-mediated effects on the migration and expression of angiogenesis biomarkers in human breast adenocarcinoma cells, Ash et al. have also documented increased migration of breast cancer cells, when exposed to 65% oxygen as compared to 25% oxygen [86]. Crowley et al. tested the effects of four oxygen concentrations, 21%, 30%, 60% and 80%, O_2_, on both ER+ and ER− breast cancer cell lines, and found that, short-term, 3 h of 60% oxygen exposure enhances migration and also promotes the secretion of several pro-metastatic angiogenesis factors such as VEGF, IL-8 and angiogenin [87].

Epithelial mesenchymal transition (EMT) is an essential cellular program, normally active during embryogenesis and wound healing. During this biological process, a normal epithelial cell, in contact with the basement membrane, turns into a mesenchymal phenotype and migrates from the original epithelial layer. The onset of EMT in cancer was associated with an increased risk of metastatic disease and cancer progression [88,89]. EMT is accompanied by increasing the expression of the mesenchymal markers (e.g., vimentin) and by decreasing the expression of the epithelial markers, such us E-Cadherin. However, in partial EMT, cells may co-express both mesenchymal and epithelial markers or may lose epithelial markers without gaining mesenchymal markers. Partial EMT plays an important role in metastasis by enhancing tumour cell plasticity [89]. The increased expression of Vimentin, a cytoskeleton protein associated with a migration phenotype, was found to be a poor prognostic marker in cancer [90,91]. The reduced expression of E-Cadherin, a cytoplasmic protein present in the epithelial cells, correlates, in an experimental setting, with an invasive phenotype [92]. Tiron et al. demonstrated that in vitro exposure of ER-breast cancer cell lines to 80% O_2_ for 6 h increases ROS and induces BDNF, VEGF-R2 and vimentin expression and thus promotes EMT and angiogenesis [93]. Tested in vivo, in a murine model of ER-breast cancer, the perioperative exposure to 80% oxygen for 6 h was associated with an increased number and size of hepatic metastasis, evaluated at 4 weeks after the surgical excision of the tumour [93].

On the other hand, Kim et al. evaluated the effects of hyperoxia exposure on a non-surgical murine model of lung cancer. The authors used 85% oxygen concentration for 24 h, a longer period than used in previous studies, and reported increased ROS activity followed by cellular apoptosis via the MAPK pathway. The exposure has been associated with decreased size and number of lung tumours [94].

The controversial results of these studies, summarized in Table 2, may be explained by the heterogeneity of the experimental designs, including the use of different cancer cell lines (types of tumour, human versus murine cancers), oxygen concentrations and exposure durations.

### 3.4. Brain-Derived Neurotrophic Factor (BDNF)

Hyperoxia increases ROS and activates cell defence systems in order to keep ROS within physiological range, as previously discussed. BDNF is one of the molecules that are upregulated in response to hyperoxia exposure, as shown in various experimental settings, including the peribronchial smooth muscle of neonatal rats [95], the Alzheimer mouse model [96], the breast cancer mouse model [93] and healthy volunteers [97]. Interestingly, some healthy volunteers enrolled in the study [97] resigned due to the side effects, although the tested oxygen concentration was only 37% O_2_. BDNF and its main receptor, tropomyosin receptor kinase B (TrkB) have been reported to promote alveolar epithelial regeneration after lung injury [98] or to exert neuroprotective effects [99,100,101,102,103,104]. However, TrkB can be activated by cyclic adenosine monophosphate [105] in order to exert the neuroprotective effects. These data suggest that TrkB plays a pivotal role in response to ROS. However, the activation signals can come either from BDNF or from other agonists, depending on cell type and ROS sub-type. The interaction BDNF-ROS does not consist only in BDNF overexpression in response to increasing levels of ROS, as it has been reported that BDNF exerts various biological effects by the generation of oxidative stress in human vascular endothelial cells [106,107].

BDNF-TrkB axis exerts various effects depending on the cell types. In normal cells, it protects cells from high levels of ROS or induces moderate ROS levels in other circumstances. In cancer cells, in addition to pro-survival signalling, the BDNF-TrkB molecular pathway induces EMT, which is associated with poor prognostic in various cancer types [108,109], may increase migration and invasion [110,111], may promote cancer cell survival and proliferation [112,113] and may increase neo-angiogenesis through increasing vascular endothelial growth factor (VEGF) expression [114,115]. The authors from [116] reported that BDNF-TrkB axis induces VEGF expression via HIF-1α. HIF-1α is generally accepted to have an increased expression in hypoxia and a decreased expression in hyperoxia conditions. Tiron et al. identified an upregulation of HIF-1α protein expression after short hyperoxic exposure of triple negative breast cancer cells in an experimental mouse model of perioperative care [93]. These data join the new sparse data that support HIF-1α increased protein expression after the exposure to hyperoxic episodes [80,117] and suggest that, in certain circumstances (e.g., mild hyperoxia), HIF-1α protein expression is not impaired as in prolonged high grade hyperoxia. In non-cancer patients, HIF-1α may have beneficial effects (e.g., muscle regeneration), but in oncologic patients it may promote cancer progression and recurrence.

Cells that are detaching by the extracellular matrix will undergo a process of cell death called anoikis. There are data [118,119,120,121] demonstrating that BDNF-TrkB axis induce anoikis resistance in various cancer types, in addition to EMT. BDNF synthesized as the result of perioperative oxygen exposure in oncological patients may promote survival of the circulating tumour cells and formation of new metastases; in addition it can decrease the time to relapse by increasing cancer cell proliferation and EMT at the level of existing micrometastases.

Chemoresistance occurs in many types of cancer in clinical setting. In breast cancer, it has been reported that multi-nucleated cells generated by chemotherapy drugs are oxidatively stressed, and this induces chemoresistance, as shown both in vitro and in vivo [122]. Moreover, the multi-nucleated cells induce chemoresistance by secreting VEGF, activating the RAS/MAPK pathway and, thus, ROS—HIF-1α signalling plays a crucial role. It could be possible that oxidatively stressed multi-nucleated cells to increase the expression of BDNF in order to counteract ROS, and BDNF would be responsible for activating the HIF-1α-VEGF pathway, which further induces chemoresistance.

Another source of ROS production in cancer is represented by ionizing radiation. Cancer radiotherapy changes the tumour microenvironment, which in some circumstances induces resistance and recurrence, as already published [123,124]. BDNF does not emerge as a key molecule in radio-resistance; however, the classical downstream signalling pathway of the receptor tyrosine kinase family is involved, as inhibition of PI3K/mTOR induced radio-sensitization in pediatric and adult glioblastoma [125]. However, it has been shown that low-level laser therapy can rescue dendrite atrophy by upregulating BDNF expression via ROS generation [126]. These data suggest that, in certain cancer types, subtypes and radiation clinical models, cancer cells are able to express enough BDNF to protect themselves against the increased ROS levels generated by radiation, the mechanism responsible in many cases for radio-resistance phenomena. On the other hand, it has been demonstrated that total abdominal irradiation causes cognitive deficits in mice models [127]. Abdominal irradiation shifted gut bacterial composition, which increased the expression of miR-34a-5p in small intestine tissue and also in the peripheral blood. Expressed miR-34a-5p targeted the 3′UTR of BDNF mRNA in hippocampus to mediate cognitive dysfunction. This mechanism was further prevented by tail intravenous injection of a miR-34a-5p antagomir. All together, these data may explain why the irradiation of some colon cancers is associated with good prognosis while, in other cases, radio-resistance occurs. Increased BDNF levels may favour radio-resistance by its pro-survival role and also by the induction of the epithelial to mesenchymal transition (EMT) process. On the other hand, a decreased BDNF level induced by irradiation and the shift in the bacterial composition of gut microbiota may sensitize the cancer cells.

### 3.5. Hyperoxic-Hypoxic Paradox

In clinical circumstances, fluctuations in oxygen levels due to hyperoxic episodes may induce the expression of many mediators usually induced by hypoxia. This phenomenon, known as the “hyperoxic-hypoxic paradox” has been recently reviewed by Hadanny and Efrati [128]. Although the hyperoxic-hypoxic paradox was identified after repeated intermittent hyperoxia, Tiron et al. [93] demonstrates the expression of HIF-1α and VGFR after a single hyperoxia exposure, factors which are normally expressed under hypoxic conditions.

While immunosuppression mediated by tumour hypoxia has been extensively investigated, the data regarding the interaction of perioperative hyperoxia with immune system is lacking. HIF-1α plays an important role in immunosuppression, mediated by chronic hypoxia. We speculate that HIF-1α expressed after acute perioperative hyperoxia may interact with the immune system, probably in a lower extend compared with chronic hypoxia.

**Figure 1:**
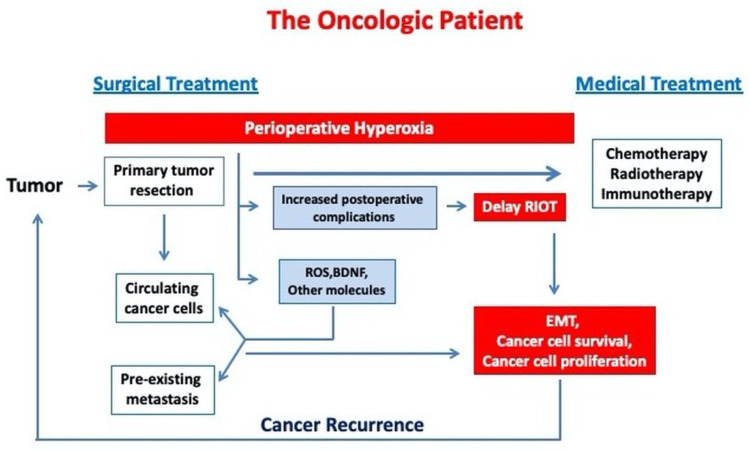
Perioperative hyperoxia may induce cancer progression in oncological patients by increasing cancer cell survival and EMT. (RIOT—return to the intended oncological treatment, EMT—epithelial mesenchymal transition).

Figure 1 describes the link between perioperative hyperoxia exposure and cancer progression in oncologic patients.

## 4. Conclusions

Hyperoxia exposure, mostly in critically ill patients but also during the perioperative period, was shown to be associated with oxidative stress, organ-specific side effects and increased mortality.

In surgical oncologic patients, increased inspiratory oxygen concentration exposure during the perioperative period can lead to increased production of the reactive oxygen species and oxidative stress, and can induce various grades of partial to full epithelial to mesenchymal transition in cancer cells. Although the primary tumours are surgically removed, the effects of hyperoxia on distal micrometastases and/or on circulating cancer cells can promote cancer progression or recurrence. The molecules that mediate the reactive oxygen species effects on cancer progression may be dependent on cancer cell type. These molecules, such as BDNF, must be upregulated in response to transient increased ROS and, at the same time, be able to induce cell survival, cell proliferation and EMT of cancer cells. EMT generates cancer stem cells, enhances cell migration and invasion and promotes cancer recurrence.

At this moment, the adequate or the safe dose of oxygen therapy in surgical cancer patients, in order to maximize beneficial and minimize harmful effects, is not clearly defined. The available evidence does not support the use of perioperative hyperoxia in major oncological surgery. The oxygen dose during the perioperative period must be tailored in order to avoid both hypoxemia and hyperoxemia.

It is important to produce new experimental and clinical data that may guide practitioners to properly use the most commonly prescribed drug in hospitalized patients.

## Figures and Tables

**Table 1:** Clinical studies exploring hyperoxia exposure effects during the perioperative period in surgical patients.

Author, Year, Reference	Study Type	Patients Number	Type of Patients	Hyperoxia Exposure	Primary Outcome	Results
Cohen, B. et al., 2019 [29]	Post-hoc analysis of [30]	5056	Colorectal surgery	Intraoperative, 39 vs. 80% O_2_	Postoperative pulmonary complications	No difference
Mattishent, K. et al., 2019 [31]	Meta-analysis	3839, 3458	Surgical patients	Perioperative, 30 vs. 80% O_2_	Postoperative atelectasis, pneumonia	No difference
Staehr-Rye, A.K. et al., 2017 [32]	Retrospective registry study	73,922	Non-cardiotoracic surgery	Intraoperative, 30%, 40%, 51%, 58%, 79% O_2_	Major respiratory complication (re-intubation, respiratory failure, pulmonary oedema, pneumonia)	Hyperoxia increased risk, in a dose dependent manner
Smit, B. et al., 2018 [33]	Meta-analysis	408 392	Healthy volunteers, medical and surgical patients	PaO_2_ = 234–617 mmHg	Hemodynamic effects	Hyperoxia decreased cardiac output, increased systemic vascular resistance
Fonnes, S. et al., 2016 [37]	Post-hoc analysis of [49]	1386	Abdominal surgery	Intraoperative, 2 h postoperative 30 vs. 80% O_2_	Long-term major cardiovascular complication	Hyperoxia (80% O_2_) increased acute coronary syndrome
Meyhoff, C. et al., 2009 [49]	RCT	1386	Abdominal surgery	Intraoperative, 2 h postoperative 30 vs. 80% O_2_	Surgical site infection within 14 days	No difference
Meyhoff, C. et al., 2012 [50]	2.3 years follow-up	1386 352	Abdominal surgery Cancer patients	Intraoperative, 2 h postoperative 30 vs. 80% O_2_	Long-term mortality	Hyperoxia increased long term mortality in cancer patients
Meyhoff, C. et al., 2014 [51]	3.9 year follow-up	1377	Abdominal surgery Cancer patients	Intraoperative, 2 h postoperative 30% vs. 80% O_2_	Risk of new or recurrent cancer at 3.9 years follow-up	Shorter cancer-free survival time
Podolyak, A et al., 2016 [52]	Follow-up [53,54]	927 (432 + 495)	Colorectal surgery	Intraoperative, 2 h postoperative 30% vs. 80% O_2_	Long-term mortality analysis	No difference
Greif, R. et al., 2000 [53]	RCT	500	Colorectal surgery	Intraoperative, 2 h postoperative 30% vs. 80% O_2_	Surgical site infection within 30 days	Decreased in hyperoxia group
Kurz, A. et al., 2015 [54]	RCT	585	Colorectal surgery	Intraoperative, 2 h postoperative 30% vs. 80% O_2_	Surgical site infection within 30 days	No difference
Kurz, A. et al., 2018 [30]	Alternating intervention controlled trial	5749	Major intestinal surgery	Intraoperative 30% and 80% O_2_, alternating at 2-week intervals for 39 months	30-day composite of deep tissue or organ-space SSI, healing-related wound complications, mortality	No difference
Jiang, Q. et al., 2021 [55]	Post-hoc analysis of [30], 3 years follow-up	2800 995	Colorectal surgery Cancer patients	Intraoperative 30% and 80% O_2_, alternating at 2-week intervals for 39 months	Long term mortality	No difference

**Table 2:** Experimental in vitro and in vivo studies exploring hyperoxia exposure effects in cancer.

Author, Year, References	Study Type	Experimental Design	Hyperoxia Exposure	Primary Outcome	Results
Li, L. et al., 2007 [56]	In vivo	C57BL/6 mice exposed to high-VT mechanical ventilation	21 vs. 95% O_2_ for 1–5 h	Ventilator-induced lung injury	Hyperoxia increases lung and inflammation ventilator-induced lung injury
Tiron, A. et al., 2020 [93]	In vitro, In vivo	MCF10A, MDA-MB-231, 4T1 breast cancer cells 4T1 TNBC Murine model	21%, 40%, 60%, 80% O_2_ for 6 h 21%, 40%, 60%, 80% O_2_ for 6 h perioperative	Effects on breast cancer growth	Hyperoxia (80%) increases ROS, BDNF, EMT and angiogenesis molecules Increases size and number of lung metastasis
Crowley et al., 2018 [87]	In vitro	MDA-MB-231 MCF-7 breast cancer cells	21%, 30%, 60%, 80% O_2_ for 3 h	Effects on breast cancer cell migration and angiogenesis	Hyperoxia (60%) promotes migration and upregulates angiogenesis factor secretion
Ash et al., 2014 [86]	In vitro	MDA-MB-231 and MCF-7 breast cancer cells	Xenon 70% + O_2_ 25%, sevoflurane 2.5% + 65% O_2_ for 5 h	Effects on breast cancer cells migration and angiogenesis	Hyperoxia (65%) promotes breast cancer cell migration
Kim et al., 2018 [94]	In vitro In vivo	A549 lung cancer cells Murine model of lung cancer	85% O_2_ for 24 h	Morphological changes in lung cancer	Hyperoxia increased ROS, apoptosis Decreases size and number of lung tumors
Qian et al., 2018 [79]	In vivo	4T1 TNBC murine model	21%, vs. 65% O_2_ for 21 days	Effects on tumor microenvironment	Hyperoxia reverses immunosuppression by regulating myeloid-derived suppressor cells and PD-L1 expression
Hatfield et al., 2015 [83]	In vitro In vivo	MCA205 tumor cell line 4T1 TNBC Murine model	40%, 60% O_2_ for 2 days	Effects on tumor microenvironment	Hyperoxia increases tumoral infiltration with CD8+ T cells, proinflammatory cytokines, decreased TGF-β and immunosuppression by T-reg cells.

## Data Availability

The data will be freely available by open access.
